# Conventional vs. Drug-Eluting Beads Transarterial Chemoembolization for Unresectable Hepatocellular Carcinoma—A Propensity Score Weighted Comparison of Efficacy and Safety

**DOI:** 10.3390/cancers14235847

**Published:** 2022-11-27

**Authors:** Lynn Jeanette Savic, Evan Chen, Nariman Nezami, Nikitha Murali, Charlie Alexander Hamm, Clinton Wang, MingDe Lin, Todd Schlachter, Kelvin Hong, Christos Georgiades, Julius Chapiro, Fabian M. Laage Gaupp

**Affiliations:** 1 Section of Vascular and Interventional Radiology, Department of Radiology and Biomedical Imaging, Yale School of Medicine, New Haven, CT 06510, USA; 2 Department of Radiology, Charité—Universitätsmedizin Berlin, Corporate Member of Freie Universität Berlin and Humboldt-Universität zu Berlin, 13353 Berlin, Germany; 3 Berlin Institute of Health at Charité—Universitätsmedizin Berlin, 10178 Berlin, Germany; 4 Division of Vascular and Interventional Radiology, Department of Diagnostic Radiology and Nuclear Medicine, University of Maryland School of Medicine, Baltimore, MD 21201, USA; 5 Experimental Therapeutics Program, University of Maryland Marlene and Stewart Greenebaum Comprehensive Cancer Center, Baltimore, MD 21201, USA; 6 Division of Vascular and Interventional Radiology, Russel H. Morgan Department of Radiology and Radiological Sciences, Johns Hopkins University School of Medicine, Baltimore, MD 21218, USA

**Keywords:** hepatocellular carcinoma, outcome, prognostic prediction, propensity score analysis, TACE, drug-eluting beads, lipiodol

## Abstract

**Simple Summary:**

Transarterial chemoembolization (TACE) is a guideline-approved, minimally invasive therapy for unresectable hepatocellular carcinoma (HCC). This study investigated the efficacy and safety of two frequently performed types of TACE that are currently used interchangeably. The statistical method of propensity score weighting was used to reduce bias in the results based on baseline differences and make the groups as comparable as possible. In a large cohort of 370 HCC patients, no significant difference in overall survival was observed between the two TACE groups and adverse advents occurred with similar frequency in both groups. However, conventional TACE showed superior efficacy in patients with infiltrative disease, whereas drug-eluting beads TACE was more effective in nodular tumors. These findings suggest that tumor morphology and distribution on baseline imaging can inform decisions on the type of TACE that the individual patient would benefit from the most.

**Abstract:**

This study compared the efficacy and safety of conventional transarterial chemoembolization (cTACE) with drug-eluting beads (DEB)-TACE in patients with unresectable hepatocellular carcinoma (HCC). This retrospective analysis included 370 patients with HCC treated with cTACE (n = 248) or DEB-TACE (n = 122) (January 2000–July 2014). Overall survival (OS) was assessed using uni- and multivariate Cox proportional hazards models and Kaplan-Meier analysis. Additionally, baseline imaging was assessed, and clinical and laboratory toxicities were recorded. Propensity score weighting via a generalized boosted model was applied to account for group heterogeneity. There was no significant difference in OS between cTACE (20 months) and DEB-TACE patients (24.3 months, ratio 1.271, 95% confidence interval 0.876–1.69; *p* = 0.392). However, in patients with infiltrative disease, cTACE achieved longer OS (25.1 months) compared to DEB-TACE (9.2 months, ratio 0.366, 0.191–0.702; *p* = 0.003), whereas DEB-TACE proved more effective in nodular disease (39.4 months) than cTACE (18 months, ratio 0.458, 0.308–0681; *p* = 0.007). Adverse events occurred with similar frequency, except for abdominal pain, which was observed more frequently after DEB-TACE (101/116; 87.1%) than cTACE (119/157; 75.8%; *p* = 0.02). In conclusion, these findings suggest that tumor morphology and distribution should be used as parameters to inform decisions on the selection of embolic materials for TACE for a more personalized treatment planning in patients with unresectable HCC.

## 1. Introduction

Transcatheter arterial chemoembolization (TACE) is recognized as a guideline-approved treatment for unresectable intermediate-stage hepatocellular carcinoma (HCC), defined as Barcelona Clinic Liver Cancer (BCLC) stage B, but also proved effective and safe in early and advanced-stage HCC [1,2]. First introduced in 1977, conventional TACE (cTACE) utilizes an emulsion of Lipiodol ^®^ (Guerbet, Villepinte, France) and chemotherapeutic agents followed by the injection of embolizing material [3]. However, varying cTACE protocols are in use to date that limit the comparability of efficacy reports from different institutions, calling for strategies to improve the standardization of the technique. Moreover, cTACE may suffer from its limited ability to release the chemotherapeutic agents in a controlled manner and possible washout of the emulsion, which may reduce the locally retained drug payload in the tumor and potentially increase non-target drug exposure [3,4]. Thus, drug-eluting beads (DEB)-TACE was developed, allowing for a more sustained chemotherapy administration over time, while theoretically decreasing off-target toxicity [5,6]. As a result, many clinical centers have adopted DEB-TACE as the de facto standard of care for the intra-arterial treatment of HCC. 

To date, several randomized controlled trials (RCT) comparing DEB-TACE and cTACE have demonstrated comparable results in tumor response and safety [6,7,8,9]. Additionally, a recent prospective trial demonstrated low systemic concentrations of doxorubicin following segmental cTACE, which were comparable to those previously reported for DEB-TACE [10]. However, numerous meta-analyses revealed prolonged overall survival (OS) in HCC patients receiving DEB-TACE compared to cTACE and fewer adverse events (AEs) [11,12,13,14,15,16,17], which was also confirmed by several more recent retrospective studies [18,19]. 

Although the literature overall suggests superiority of DEB-TACE over cTACE, there is no definite consensus as to which technique should be used for the treatment of unresectable HCC [1,20]. Consequently, DEB-TACE and cTACE are currently often used interchangeably in clinical practice. Therefore, an unmet clinical need for biomarkers exists that predict tumor susceptibility to one TACE technique or the other, which could allow for a more personalized treatment planning and improved clinical outcome. Thus, this study aimed to compare the OS and AEs and identify prognostic factors in patients with HCC treated with either cTACE or DEB-TACE using propensity score weighting. 

## 2. Materials and Methods

### 2.1. Study Cohort

**Figure 1:**
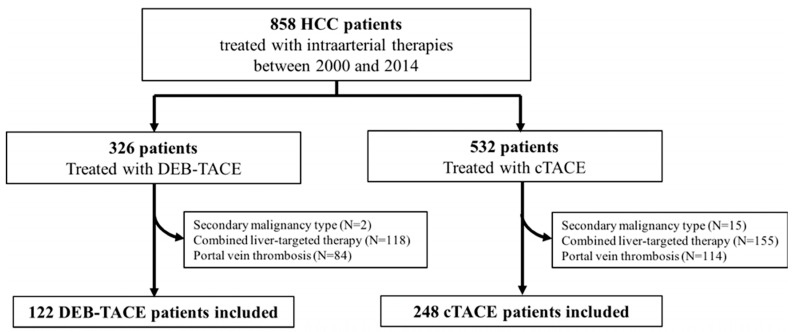
Flowchart of patient selection. HCC, hepatocellular carcinoma; cTACE, conventional transarterial chemoembolization; DEB-TACE, drug-eluting beads TACE; PVT, portal vein thrombosis.

Institutional review board (IRB) approval was obtained, and Health Insurance Portability and Accountability Act (HIPAA) regulations were followed for this retrospective single-institution study. Informed consent was waived. Between January 2000 and July 2014, 858 patients with HCC were treated using TACE. We have previously reported on HCC patients with PVT treated with cTACE or DEB-TACE (BLINDED) [21]. These patients were excluded from the current analysis. HCC was diagnosed on imaging or histopathology [22]. Inclusion and exclusion criteria are listed in the study flowchart (shown in Figure 1). Patients who had received loco-regional treatments of the same lesions prior to TACE or who received combined locoregional therapies (e.g., TACE and ablation) were excluded. 

While our study includes patients that had TACE between 2000 and 2014, DEB-TACE was only introduced in 2006. Therefore, between 2000 and 2005, patients were exclusively treated with cTACE, while between 2006 and 2014, cTACE and DEB-TACE were used in parallel at this institution. During this period, no definite institutional algorithm governed the choice of cTACE vs. DEB-TACE. The choice of treatment was decided on a case-by-case basis within the interdisciplinary tumor board and by discussion with the patient and informed consent. To account for possible differences in treatment protocols and selection bias after the introduction of DEB-TACE at our institution, we first compared the cohort receiving cTACE prior to 2006 (n = 122) to the cohort receiving cTACE after 2006 (n = 126). Next, those treated with cTACE following introduction of DEB-TACE (2006) were compared with those treated with DEB-TACE. Finally, the complete cohort of cTACE patients (2000–2014) was compared to the DEB-TACE cohort (2006–2014).

Overall, we included 248 cTACE patients and 122 DEB-TACE patients who had received a total of 781 TACE procedures (shown in Figure 1). Mean treatment sessions were 2.3 ± 0.1 in the cTACE group (range, 1–10 sessions) and 1.7 ± 0.1 (range, 1–6 sessions) in the DEB-TACE group. Baseline patient characteristics are listed in Table 1, and baseline tumor characteristics are listed in Table 2. 

To further investigate the difference in cTACE and DEB-TACE efficacy in patients with specific HCC characteristics and to identify possible prognostic biomarkers, the patients were further stratified according to a) tumor morphology into groups with infiltrative (n = 83) or nodular disease (n = 287) and b) tumor size into groups with dominant lesions larger than 3 cm (n = 269) or larger than 5 cm (n = 167). Dominant lesions were defined as the largest HCC lesion. The size cutoff values were adopted from the BCLC staging system (3 cm) and the Milan criteria (5 cm) used for transplantation candidates [1,23]. Macroscopic growth patterns were defined based on the three pathological categories: nodular, massive, and infiltrative [24]. Specifically, infiltrative tumors were defined as diffuse, ill-defined lesions on baseline MRI as opposed to nodular HCC with clear demarcation and massive HCC occupying most or all of a hepatic lobe [25]. In this study, patients with nodular disease included those with nodular and massive HCC subtypes. Additionally, morphological features of cirrhosis were assessed on MRI (e.g., liver contour, differences in signal intensities on in- and opposed phase T1-weighted sequences).

### 2.2. Transarterial Chemoembolization Techniques

All hepatic interventions were performed or supervised by three experienced interventional radiologists with 10, 15, and 21 years of experience. Naturally, experience levels have further evolved over time given the relatively long study duration of 14 years. A consistent approach was used for all patients according to IRB-approved standard institutional protocol. Prior to the procedure, hepatic arterial anatomy, tumor vascularity and portal venous patency were determined on angiographic exams. cTACE was done via lobar or segmental (selective) or subsegmental (superselective) injections. A 1:1 oil-drug emulsion was made by mixing 10 mL of Lipiodol with 10 mL of 0.9% saline containing 50 mg of doxorubicin and 10 mg of mitomycin C. The administered volume of the chemoemulsion was titrated to the tumor volume. Next, embolic microspheres (Embospheres, Merit Medical, South Jordan, Utah) of 100–300 μm or 300–500 μm diameter were injected to achieve significant flow reduction to the tumor, which was used as the endpoint of embolization. 

For DEB-TACE, selective embolization was performed with 100–300 μm diameter LC Beads (Biocompatibles/BTG, Surrey, United Kingdom). Up to 4 mL of drug-eluting beads were loaded by mixing them with 25 to 37.5 mg of doxorubicin per mL. No more than 100 mg of doxorubicin was administered per session. Complete occlusion of the tumor-feeding blood vessels was avoided, maintaining patency of the tumor-feeding arteries for potential repeat treatments. In case of persistent tumor hypervascularization after TACE or incomplete first treatment, therapies were repeated with the same TACE modality as the initial treatment. If there was no treatment response or disease progression was observed on follow-up, magnetic resonance imaging (MRI) with contrast was performed. 

### 2.3. MRI and Image Analysis

All patients underwent imaging with a standardized contrast-enhanced liver MRI protocol prior to the initial TACE procedure. The protocol included unenhanced and contrast-enhanced (0.1 mmol/kg intravenous gadopentate; Magnevist; Bayer, Wayne, NJ, USA) sequences in the hepatic arterial phase (20 s after contrast administration), portal venous phase (70 s after contrast administration), and delayed phase (180 s after contrast administration). Image evaluation of tumor morphology and caliper-based diameter measurements were retrospectively performed by two independent 4th year radiology residents and supervised by a board-certified radiologist. Discrepancies were solved by consensus.

### 2.4. Overall Survival

The primary endpoint of this study was OS, defined as time from first TACE (study entry point) until death due to any cause. Follow-up ended on 31 December 2014. Patients who received liver-targeted therapies other than TACE, a different form of TACE than was initially performed, or liver transplantation following initial TACE were censored at time of additional therapy. 

### 2.5. Adverse Events

The secondary endpoint of this study was evaluation of treatment-related toxicity. Of all patients receiving cTACE, 157 and 125 patients had post-procedural reports for clinical AEs and biochemical toxicity, respectively. Of all patients receiving DEB-TACE, clinical AEs and biochemical toxicity reports were available in 116 and 115 patients, respectively. Clinical AEs and biochemical toxicities were recorded for individual DEB-TACE and cTACE procedures. Treatment-related AEs and biochemical toxicities occurring within 30 days after TACE were included into the analysis. AEs were graded by National Cancer Institute Common Terminology Criteria for Adverse Events (CTCAE, v4.03). Biochemical toxicities of ≥Grade 3 were reported. 

### 2.6. Statistical Analysis

Descriptive statistics were reported as mean ± standard deviation or median and range. Using propensity score matching a balanced dataset of observed covariates across both treatment modalities was generated. Baseline characteristics utilized for BCLC classification were used as covariates, and gradient boosting was used to obtain propensity scores. Patients of one group were matched with one or more patients of the comparison group on propensity score. The scores were weighted, including all individuals in the analysis. To confirm comparability of both cohorts after propensity score weighting, baseline patient and tumor characteristics were compared using contingency tables and the Fisher’s exact test (two parameters) or Chi square test (>2 parameters), respectively. A propensity score-weighted Cox proportional hazards regression model was fitted to identify predictors of OS. Covariates that were significantly correlated with OS in univariate analysis were included in multivariate analysis. Additionally, time-to-event data was analyzed by plotting Kaplan-Meier curves using weighted outcomes and comparing survival curves by the log-rank test (OS, ratio, 95% confidence interval of ratio). Comparison of post-TACE AEs was conducted using Fisher’s exact test. The complete statistical analysis was conducted in R 3.0.3 (2014, R Core Team, Vienna, Austria). The add-in R package “twang” was used to perform propensity score weighting, and the “survival” package was used for survival analysis. *p*-values < 0.05 were considered statistically significant.

## 3. Results

### 3.1. Identification of Predictors of the Overall Survival after TACE

#### 3.1.1. Propensity Score Weighting

A good balance was achieved in the covariates following propensity score weighting. The standardized absolute difference in means for all covariates was below 0.05, which is lower than the recognized threshold value of 0.2. A sufficiently low standardized difference in means indicates that the distribution of baseline covariates is independent of treatment assignment after propensity score weighting. When comparing baseline patient and tumor characteristics of both treatment groups after propensity score weighing (Table 1 and Table 2), no significant differences were observed except for the presence of cirrhosis (*p* = 0.018). However, individual Child-Pugh scores were comparable in both cohorts (*p* = 0.557).

#### 3.1.2. Multivariate Cox Proportional Hazards Regression

The median OS of the entire cohort was 28.1 months. During the observation period, 215 patients had died. There was no significant effect on survival whether patients were treated with DEB-TACE or cTACE prior to adjustment for other potential confounding covariates. This was also true after adjusting for covariates significant in univariate analysis, although DEB-TACE exhibited slightly better outcomes compared to cTACE (*p* = 0.61, HR = 0.92). After propensity score weighted analysis, Child-Pugh classes B + C vs. A (*p* ≤ 0.001; HR = 2.03), dominant lesion size greater than 3 cm (*p* ≤ 0.001; HR = 2.34), the presence of tumor multiplicity (*p* = 0.014; HR = 1.52), extrahepatic metastases (*p* = 0.010; HR = 1.77), and infiltrative tumor status (*p* ≤ 0.001; HR = 1.76) were found to independently predict patient survival (Table 3).

### 3.2. Survival Analysis

#### 3.2.1. cTACE before vs. cTACE after the Introduction of DEB-TACE in 2006

There was no significant difference in survival comparison for patients treated with cTACE before and after 2006 prior to adjustment (*p* = 0.406). Even after propensity score weighting, no difference was observed in comparing cTACE before and after the introduction of DEB-TACE. Median OS was 17.1 months (95% CI, 14.1–23.2) before and 19.0 months (95% CI, 15.5–25.1) after 2006, respectively (*p* = 0.665). 

#### 3.2.2. cTACE vs. DEB-TACE after the Introduction of DEB-TACE in 2006

When comparing survival in cTACE patients treated after 2006 with the DEB-TACE cohort without propensity score weighting, there was no significant difference observed in OS (*p* = 0.352). Propensity score adjustment comparison also failed to demonstrate a significant difference; cTACE and DEB-TACE patients had a median OS of 21.2 months (95% CI, 18.0–28.4) and 22.6 months (95% CI, 18.8–29.1), respectively (*p* = 0.348). 

#### 3.2.3. cTACE vs. DEB-TACE without Timeframe Restriction

**Figure 2:**
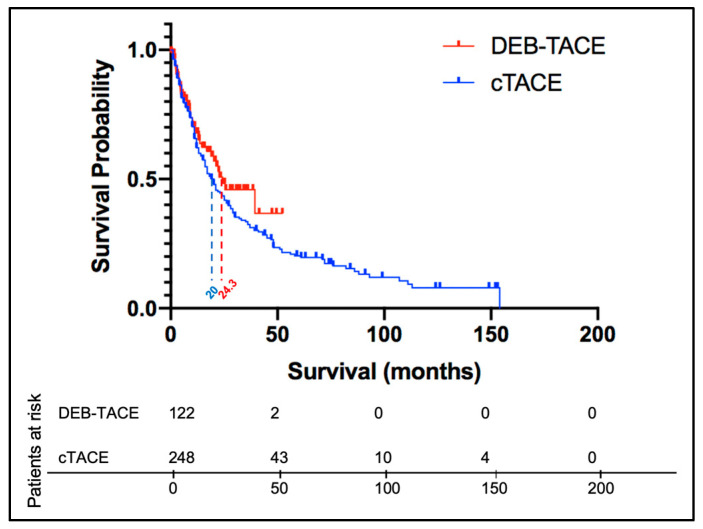
Kaplan-Meier curves demonstrating survival after propensity score weighting. Patients who received other combined liver-targeted therapies or liver transplantation after TACE were censored. Censored patients as well as those still alive at the end of the observation period are indicated in the survival curves as crosses (+). The median survival of the cTACE and DEB-TACE group was 20 months and 24.3 months (ratio 1.271, 95% confidence interval 0.876–1.69), respectively (*p* = 0.392). DEB-TACE, drug-eluting beads TACE; cTACE, conventional transarterial chemoembolization.

Propensity score weighting for the entire cTACE cohort (2000–2014) and the DEB-TACE cohort (2006–2014) revealed no significant difference in outcome with a median OS of 20 months and 24.3 months (ratio 1.271, 95% CI 0.876–1.69) after cTACE and DEB-TACE, respectively (*p* = 0.392) (shown in Figure 2). 

#### 3.2.4. Subgroup Survival Analysis: cTACE vs. DEB-TACE without Timeframe Restriction

When performing subgroup survival analysis according to dominant lesion size of 3 cm or greater, no significant difference (*p* = 0.564) was found in median OS after cTACE (16.4 months; 95% CI, 13.2–20.3) vs. DEB-TACE (13.6 months; 95% CI, 11.7–24.3). When only including patients with dominant lesions of 5 cm or greater, a slightly longer median OS of 13.1 months (95% CI, 11.4–17.1) was observed after cTACE as compared to 11.7 months (95% CI, 11.4–13.6) after DEB-TACE, which was also not significant (*p* = 0.499). 

**Figure 3:**
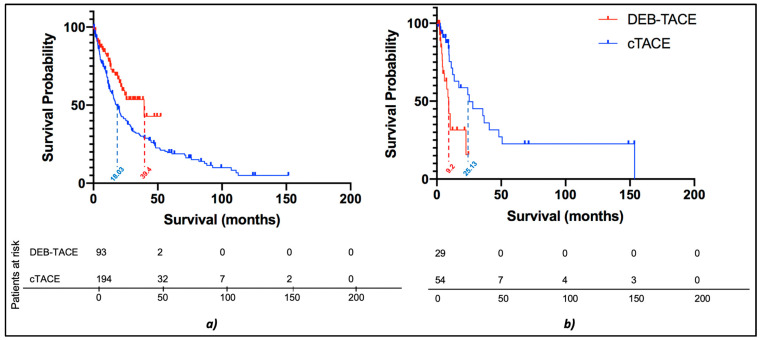
Survival comparison of cTACE and DEB-TACE among patients with nodular HCC and infiltrative HCC. (**a**) In nodular HCC, the median overall survival (OS) of the cTACE and DEB-TACE group were 18.03 and 39.4 months (ratio 0.458, 0.308–0681), respectively (*p* = 0.007). (**b**) In infiltrative HCC, the median OS of the cTACE and DEB-TACE group were 25.13 months and 9.2 months (ratio 0.366, 0.191–0.702), respectively (*p* = 0.003). Hepatocellular carcinoma, HCC; DEB-TACE, drug-eluting beads TACE; cTACE, conventional transarterial chemoembolization.

**Figure 4:**
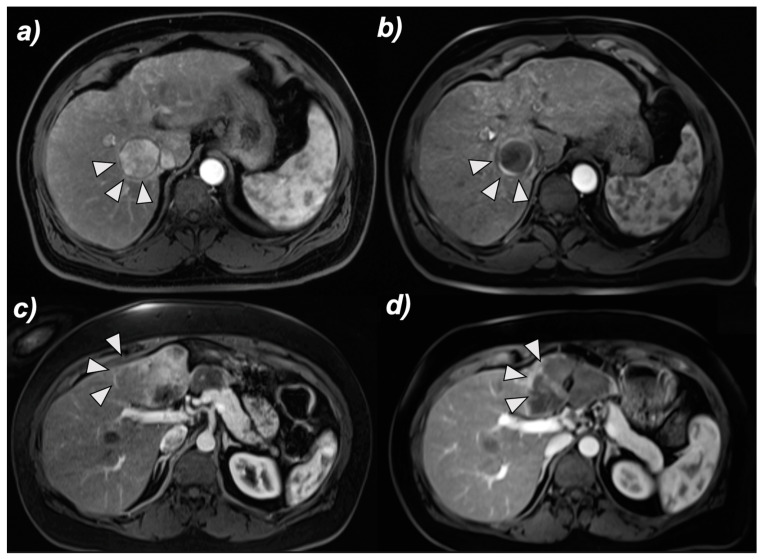
Imaging-based response of nodular and infiltrative HCC to TACE. Representative contrast-enhanced MR images of a nodular HCC treated (**a**) with DEB-TACE (upper row) and an infiltrative HCC (**c**) treated with cTACE (lower row), both showing good local tumor response on follow-up MRI after 4 weeks (**b**,**d**). Arrowheads indicate tumors on arterial-phase MRI scans. Hepatocellular carcinoma, HCC; DEB-TACE, drug-eluting beads TACE; cTACE, conventional transarterial chemoembolization.

While tumor size did not demonstrate any significant differences in OS when comparing cTACE and DEB-TACE, significant differences were observed when comparing these treatment modalities in patients stratified according to tumor morphology (infiltrative vs. nodular disease) on baseline imaging. In patients with infiltrative disease, the median OS was 25.13 months when treated with cTACE, as compared to 9.2 months (ratio 0.366, 0.191–0.702) when treated with DEB-TACE, which demonstrated a significant difference in favor of cTACE (*p* = 0.003, shown in Figure 3b). Conversely, in nodular disease, survival was significantly prolonged in patients receiving DEB-TACE as compared to patients receiving cTACE with a median OS of 18.03 months vs. 39.4 months (ratio 0.458, 0.308–0681), respectively (*p* = 0.007, shown in Figure 3a and Figure 4).

### 3.3. Adverse Events

Clinical AEs and biochemical toxicities are reported in Table 4. The incidence of observed clinical toxicities showed no significant differences except for abdominal pain/discomfort, which occurred more frequently after DEB-TACE (*p* = 0.02). Further, no significant differences in biochemical toxicities indicating liver function impairment after treatment were observed between cTACE and DEB-TACE. The most common AEs occurring after DEB-TACE and cTACE, respectively, were as follows: abdominal pain/discomfort (101/116 (87.1%) and 119/157 (75.8%)), nausea/vomiting (75/116 (64.7%) and 89/157 (56.7%)), and fatigue (8/116 (6.9%) and 19/157 (12.1%)) (Table 4). 

## 4. Discussion

The main finding of this study using propensity scoring and multivariate regression modeling is that cTACE demonstrated superior efficacy in patients with infiltrative HCC, whereas DEB-TACE was more effective in nodular HCC. Overall, the comparative analysis did not identify statistically significant survival benefits of one technique over the other with an overall favorable toxicity profile of both TACE approaches. 

The equivalent clinical outcomes reported for cTACE and DEB-TACE in this study are in agreement with available literature that remains controversial as to which technique is superior. Drug-eluting beads were commercially introduced in 2006, and since then, DEB-TACE has become the de facto standard in many clinical centers worldwide bolstered by two retrospective studies [26,27]. However, the first study only compared small and differently sized groups enrolled in different periods without correction of confounding factors [26]. The second study did not utilize OS as a primary endpoint, and the survival analysis after the first year of follow-up was predicted on a low volume of cases at risk [27]. Although the current review of treatment efficacy tends to be in favor of DEB-TACE, numerous studies have demonstrated that theoretical advantages conferred by DEB-TACE do not translate to clinical improvements in survival rates, tumor response, length of post-procedural stay, and prevalence of treatment-related toxicity, including the RCT PRECISION V trial, the Precision Italia trial, and a study conducted by Sacco et al. [6,7,9].

Hence, studies have begun focusing on comparing the efficacy of TACE modalities in sub-groups of patients with specific clinical characteristics claiming that the mechanistic differences in cTACE and DEB-TACE may be advantageous in certain patients, emphasizing the need for personalized treatment planning. In this study, OS was analyzed in subgroups of patients stratified according to tumor size. Because DEB-TACE, in theory, offers more targeted delivery and controlled release, it is believed to be more effective and safer in larger tumors requiring greater chemotherapeutic doses [6]. In addition, Kim et al. showed that the presence of tumors >5 cm was a significant predictor of decreased OS in cTACE patients, which gave room for speculation that DEB-TACE is more suitable for patients with large tumors [28]. However, this study did not reveal a significant difference in the median OS after cTACE and DEB-TACE in patients with dominant lesions >3 and >5 cm (*p* = 0.564 and *p* = 0.499 respectively). These findings are consistent with a recent study demonstrating the lack of significant difference in tumor response, OS, and time to progression between cTACE and DEB-TACE in patients with tumors >5 cm [29]. Moreover, our findings concurred with the study by Vesselle et al. reporting a significant drop-off in tumor response to DEB-TACE in tumors >5 cm, which suggests the inability of either technique to effectively control disease progression of large lesions [30].

As opposed to tumor size, our study suggests that tumor morphology prior to treatment (nodular vs. infiltrative disease) is a determinantal factor when choosing the TACE modality for each individual patient. cTACE resulted in greater OS than DEB-TACE (25.13 vs 9.2 months) in infiltrative tumors, whereas DEB-TACE performed better in nodular subtype HCC (39.4 cs. 18.03 months). Due to the liquid nature of Lipiodol and its increased dispersion, cTACE may allow for greater coverage of widespread infiltrative disease. As several studies have also concluded that cTACE can be safely used in patients with infiltrative disease, cTACE is suggested to be the preferable TACE modality in patients with infiltrative HCC [31,32]. Conversely, DEB-TACE might be more successful in well-delineated HCC subtypes because these tumors have a more distinct vascular supply. They can therefore be more selectively targeted, amplifying the effect of sustained local release of chemotherapeutic agents. Additionally, DEB-TACE may also be more successful in tumors with clear vascularization due to a greater embolic effect from larger bead sizes. To our knowledge, the OS of patients with cTACE and DEB-TACE has not previously been compared in subgroups of infiltrative or nodular HCC and prospective studies are necessary to further validate these findings.

This study included patients with different BCLC stages and investigated baseline characteristics utilized for BCLC classification as covariates in the propensity score weighted analysis. Due to the exclusion of patients with PVT in this study and only few patients with extrahepatic spread, patients were mainly classified as BCLC C due to their performance status. For advanced HCC, the role of TACE has not been fully established but the recently published BCLC update recommends TACE for BCLC C patients despite the emerging role of immunotherapies in HCC [1]. A study by Gorodetski et al. examined whether either TACE modality is superior in patients with PVT but failed to demonstrate a significant advantage of either modality in this patient subset [21].

With regard to toxicity in this study, no significant differences in the prevalence of clinical and biochemical AE were seen within 30 days after the TACE procedures, except with regard to complaints of abdominal pain/discomfort, which were more frequent in patients receiving DEB-TACE. This might be because DEB-TACE is oftentimes chosen over cTACE for the treatment of sub-capsular lesions at our institution, where ablations are technically challenging. Moreover, the maximum administered drug dose is higher for DEB-TACE than cTACE, and the embolizing potential of DEB-TACE beads is greater than Lipiodol and embolic spheres utilized for cTACE. While the incidence of abdominal pain is greater after DEB-TACE, patients with DEB-TACE received a significantly lower number of total mean TACE sessions (2.3 vs. 1.7, respectively). This result has also been observed in another study comparing TACE effectiveness [33]. As it is still controversial as to which TACE technique is superior, these results can contribute to clinical decision-making, as patients who receive a lower number of treatment sessions have fewer procedure-associated complications. 

Within DEB-TACE, different bead sizes are available with varying efficacy and safety profiles. DEBs used in this study were of 100–300 μm or 300–500 μm diameter. Studies investigating the effects of 100–300 μm beads for the treatment of HCC prior to liver transplantation reported improved efficacy of doxorubicin-loaded DEBs compared to bland embolization [34] with the anthracycline distributing radially around bead-occluded vessels inducing gradually increasing tumor necrosis within 2–4 weeks [35]. While smaller beads seem to be more frequently used nowadays, there is no consensus on the optimal particle size for DEB-TACE [36]. Several studies reported good outcome in terms of tumor response, PFS, and OS for DEBs <100 μm [37,38,39] and >100 μm [19] in individual single-arm prospective and retrospective studies in HCC patients. While a recent retrospective comparison of DEB-TACE with 70–150 μm DEBs or 100–300 μm DEBs in HCC patients reported no significant differences in tumor response or AEs [40], prior comparative studies demonstrated favorable efficacy in terms of tumor response and similar or improved OS and PFS after smaller-caliber DEB-TACE compared to medium- or large-size DEBs in HCC patients [41,42]. 

However, the primary concern regarding small-caliber DEBs in HCC is the increased risk of non-target embolization, biliary damage, or postembolization syndrome. The sequential use of DEB-TACE with 70–150 μm DC beads and 100–300 μm DC beads was associated with greater hepatobiliary toxicity than the repeated use of 100–300 μm DC beads, with no difference in tumor response [43]. A translational study in the VX2 rabbit tumor model recently showed that both 40 and 100 μm idarubicin-eluting Oncozene**^®^** microspheres demonstrated significant anti-tumoral effects, but suggested a higher risk and toxicity profile of the smaller embolics due to the differences in depth of vascular penetration, extra-tumoral deposition, extent of hypoxia, and tissue toxicity [44]. 

There are some limitations to this study. First, as a single-institution retrospective study, there was a limited pool of patients restricting the applicability of statistical analysis results beyond this cohort. Selection bias may confound the results of this comparison due to a non-randomized cohort. However, propensity score weighting was utilized to minimize possible confounders by weighting patients according to baseline characteristics. This approach also addresses the heterogeneity of the cohort and underscores the robustness of the data set representative of a realistic patient cohort that may facilitate the translation of the findings into clinical routine. Secondly, this study has a recruitment time of greater than 14 years during, which a significant number of adjustments and advancements in embolization techniques have occurred. While we have demonstrated the lack of a significant difference between cTACE before and after the introduction of DEB-TACE, slight differences in treatment techniques over time may still impact the comparison of treatment methods. Specifically, the usage of different embolic materials (e.g.**,** gelfoam) instead of particles may cause different extents of ischemia and thus affect the oncologic outcome. Finally, we have excluded patients with PVT in this study due to previous reporting. Therefore, our cohort of TACE patients is not completely representative of all HCC patients receiving TACE. This likely also explains the slightly elevated OS of the cohort as compared to current available literature. 

## 5. Conclusions

In conclusion, this study utilizing propensity score weighted analysis did not reveal a significant difference in OS of patients receiving cTACE vs. DEB-TACE for a large cohort of unresectable HCC. However, subgroup analysis of the cohort revealed that cTACE showed superior efficacy in patients with infiltrative disease, whereas DEB-TACE was more effective in nodular tumors. Thus, tumor morphology and distribution on baseline imaging may serve as imaging markers to inform decisions on the selection of embolic materials for patients undergoing TACE.

## Figures and Tables

**Table 1:** Baseline patient characteristics.

Parameter	n	n	*p*-Value
Treatment Demographics	DEB-TACE	cTACE	
	122	248	
Age			0.577
>65	49 (40.2%)	108 (43.5%)	
≤65	73 (59.8%)	140 (56.5%)	
Gender			0.285
Male	91 (74.6%)	198 (79.8%)	
Female	31 (25.4%)	50 (20.2%)	
Staging System BCLC			0.781
A	34 (27.9%)	60 (24.2%)	
B	28 (23%)	62 (25.0%)	
C	55 (45.1%)	109 (44.0%)	
D	7 (5.7%)	17 (6.8%)	
ECOG PS			0.869
0	69 (56.6%)	129 (52.0%)	
1	48 (39.3%)	107 (43.1%)	
2	4 (3.3%)	10 (4.0%)	
3	1 (0.8%)	2 (0.9%)	
Child Pugh class			0.557
A5	49 (40.2%)	86 (34.7%)	
A6	25 (20.5%)	69 (27.8%)	
B7	17 (13.9%)	35 (14.1%)	
B8	15 (12.3%)	30 (12.1%)	
B9	10 (8.2%)	13 (5.3%)	
C10	4 (3.3%)	10 (4.0%)	
C11	1 (0.8%)	3 (1.2%)	
C12	1 (0.8%)	0 (0.0%)	
C13	0 (0.0%)	2 (0.8%)	

DEB-TACE, drug-eluting beads trans-arterial chemoembolization; cTACE, conventional trans-arterial chemoembolization; BCLC, Barcelona Clinic Liver Cancer; ECOG PS, Eastern Cooperative Oncology Group performance status; SD, standard deviation.

**Table 2:** Baseline tumor characteristics.

Parameter	n	n	*p*-Value
Treatment	DEB-TACE	cTACE	
Demographics	122	248	
Size of the dominant lesion (Diameter)			0.667
>3 cm	78 (63.9%)	191 (77.0%)	
>5 cm	52 (42.6%)	115 (46.4%)	
Tumor multiplicity			0.818
unifocal	42 (34.4%)	90 (36.3%)	
multifocal	80 (65.6%)	158 (63.7%)	
Extrahepatic metastasis *			0.114
yes	1 (0.8%)	11 (4.4%)	
no	121 (99.2%)	237 (95.6%)	
Cirrhosis **			0.018
present	82 (67.2%)	134 (54.0%)	
absent	40 (32.8%)	114 (46.0%)	
Tumor type			0.895
infiltrative	29 (23.8%)	54 (21.8%)	
nodular	93 (76.2%)	194 (78.2%)	

DEB-TACE, drug-eluting beads trans-arterial chemoembolization; cTACE, conventional trans-arterial chemoembolization; SD, standard deviation. * any extrahepatic site; ** according to MRI assessment (e.g., liver contour, in- and opposed phase signal intensities, morphological appearance of liver parenchyma).

**Table 3:** Cox proportional hazard regression analysis for overall survival after propensity score weighting.

Parameter	Univariate Analysis Hazard Ratio (95% CI)	*p*-Value	Multivariate Analysis Hazard Ratio (95% CI)	*p*-Value
Treatment		0.61		
DEB-TACE	1.00			
cTACE	1.09 (0.77–1.52)			
Child Pugh Score		<0.001		<0.001
A	1.00		1.00	
B-C	2.20 (1.64–2.95)		2.03 (1.47–2.80)	
ECOG PS		<0.001		0.074
0	1.00		1.00	
>0	2.03 (1.52–2.75)		1.34 (0.97–1.84)	
Cirrhosis		0.66		
No	1.00			
Yes	0.93 (0.69–1.26)			
Size of the dominant lesion		<0.001		<0.001
≤3 cm	1.00		1.00	
>3 cm	2.29 (1.60–3.29)		2.34 (1.60–3.43)	
Number of lesions		0.002		0.014
Unifocal	1.00		1.00	
Multifocal	1.67 (1.06–2.31)		1.52 (1.09–2.12)	
Extrahepatic metastases		<0.001		0.010
No	1.00		1.00	
Yes	1.98 (1.33–2.94)		1.77 (1.14–2.74)	
Infiltrative		<0.001		<0.001
No	1.00		1.00	
Yes	2.23 (1.60–3.09)		1.76 (1.22–2.53)	

DEB-TACE, drug-eluting beads trans-arterial chemoembolization; cTACE, conventional trans-arterial chemoembolization; ECOG PS, Eastern Cooperative Oncology Group performance status.

**Table 4:** Summary of the most prevalent grade 3 or higher adverse events according to CTCAEv4.03.

	*n*	*n*	*p*-Value
Treatment	DEB-TACE	cTACE	
Adverse event	*n =* 116	*n =* 157	
Abdominal pain/discomfort	101 (87.1%)	119 (75.8%)	0.02
Fatigue	8 (6.9%)	19 (12.1%)	0.16
Nausea/vomiting	75 (64.7%)	89 (56.7%)	0.18
Fever	7 (6.0%)	4 (2.5%)	0.15
Diarrhea	4 (3.4%)	3 (1.9%)	0.44
30-day mortality	4 (3.4%)	10 (6.4%)	0.27
Biochemical toxicity	*n =* 115	*n =* 125	
Albumin	1 (0.9%)	1 (0.8%)	0.93
Bilirubin	7 (6.1%)	13 (10.4%)	0.23
ALP	3 (2.6%)	0 (0.0%)	0.07
ALT	4 (6.1%)	11 (8.8%)	0.43
AST	11 (12.2%)	18 (14.4%)	0.13

Adverse events are defined as events occurring within 30 days from the procedure reported in post-embolic follow-up visits or reports. CTCAEv4.03; common terminology criteria for adverse events version 4.03; ALP, alkaline phosphatase; ALT, alanine transaminase; AST, aspartate transaminase.

## Data Availability

The data that support the findings of this study are not publicly available to ensure that the privacy of the research participants is not compromised. However, the data is available from the corresponding author upon reasonable request.
